# 

**Published:** 1894-07-28

**Authors:** 


**Notices of Births, Deaths, and Marriages** are inserted in this column at a charge of 2s. 6d. for an announcement not exceeding four lines.

**Notice to Advertisers and Subscribers.**

All Advertisements, Orders for Copies of The Hospital, and Business Communications should be addressed to **The Publisher, NOT TO THE EDITOR**, The Hospital, 428, Strand, W.C.

The Cost of Subscription, post free, is as follows:—Per week, 2½d.; per quarter, 2s. 8d.; per half-year, 5s. 3d.; per annum, 10s. 6d Foreign:—Per Annum, 12s. 8d.; payable, in all cases, in advance.

**NOTICE TO CORRESPONDENTS.**

All MS., Letters, Books for Review, and other matters intended for the Editor should be addressed The Editor, The Lodge, Porchester Square, London, W.

The Editor cannot undertake to return rejected MS., even when accompanied by stamped directed envelope.

**"Burdett's Hospital Annual," 1894.**

Fifth year of publication. 900 pp. Boards, gilt lettering. A most complete guide to British, Colonial, Indian, and American Hospitals, Dispensaries, Nursing and Convalescent Institutions, and Asylums. Price 5s. Published by The Scientific Press (Limited), 428, Strand, W.C.

**NOTICE.—The Index for Vol. XV. (ending March 31st, 1894) is on sale at the Office of this Journal, price 2½d., post free.**

### The Chelsea Hospital for Women.

### Scraps and Gleanings.

The Hull Infirmary succeeded in collecting more than the £500 they had asked for from the Saturday Hospital Fund.

A suggestion has been made that the pedestals of old pillar letter-boxes should be used as hospital collection boxes.

Efforts are being made to raise the sum of £1,500 in order to erect an entirely new building for the Brentwood Cottage Hospital.

Last week 250 little cripples were taken for a trip to Bushwal by Mr. E. L. Boyer, the establisher of the Drift Children's Mission.

The Edinburgh Royal Infirmary has received a legacy of £1,000 by the will of the late Mr. James Crawford, of Tantallon Place, Edinburgh.

The committee appointed to inquire into the management of the Chelsea Hospital for Women report the mortality returns as being unduly high.

The results of analysing 182 samples of London water during the last month from the mains supplied by the Thames and the Lea show that the quality is excellent.

A conference of medical officers of health and sanitary inspectors has been held at Springfield Gardens to discuss means for preventing the spread of disease by vagrants.

At the quarterly meeting of the Oldham Infirmary a cheque was presented to the Rev. R. M. Davies in acknowledgment of his successful labours in Oldham for 50 years.

By the arrangement of the board of management of the Derbyshire Hospital for Children, the Hospital Thursday Committee and their wives had their annual trip early this month.

The annual sale of work in aid of the Robinson Kay Hospital for Incurables was held recently at Walmersley. There was a large gathering, and the proceeds were most satisfactory.

At the instance of the Sanitary Committee it has been decided to take the necessary steps with a view to introducing a Bill in Parliament to obtain power to provide baths and wash-houses in the City.

An appeal is made for £7,000 on behalf of the Western Ophthalmic Hospital, to enable the committee to complete the purchase of a freehold site in order to replace the present inadequate building.

The famous vine at Hampton Court Palace is heavily laden with fruit this year. There are more than 1,200 bunches of grapes. Most of it will be sent as a present from the Queen to the London hospitals.

The cases of cholera in St. Petersburg are supposed to have been caused by the reckless drinking of the poisonous water of the canal. Every street is now provided with filters and barrels of boiled water.

A drawing-room meeting was held last week at the house of the Hon. Mrs. Athelstan Riley in aid of the North-Eastern Hospital for Children The finances were shown to be in a very precarious condition, the debt having amounted to over £4,000.

The Bishop of Derry stated in his sermon at St. Peter's, Cornhill, in aid of the City of London Truss Society, that since the existence of the society no less than 50,000 persons of both sexes had obtained relief. A liberal offertory was the result of the bishop's appeal.

An anti-hydrophobic Pasteur Institute has been established in Tunis. This is the only institute of the kind in Africa at present. More than thirty-seven persons were bitten by mad dogs in Tunisia last year, several of whom were sent all the way to Paris to be treated.

The annual Sunday gathering in aid of the Leeds Infirmary and Pontefract Dispensary was a great success this year. During the course of the proceedings the suggestion was made and favourably received to found a cottage hospital in the vicinity of Leeds.

The Coombe Lying-in Hospital at Dublin have just held their annual meeting of friends and supporters. It was stated that the government had daily had to refuse applicants for admission from lack of space, and there is urgent need for extension and development.

By the will of the late Major William Hammer the residue of the funds left by him at the London and Westminster Bank, after fulfilling several charitable bequests, are to be equally divided between the hospitals of Charing Cross, St. George's, and Brompton (consumption).

A special court of the governors of the Brighton Hospital for Women has been held to consider a recommendation by the committee as to the necessity of rebuilding the out-patients' department. It was stated that the present building was obsolete and the space insufficient.

The annual general meeting of Wellington Cottage Hospital was held recently. The balance-sheet for the last year showed a deficit of £100. It was decided to hold a bazaar, by the proceeds of which it was hoped that the debt would be cleared and the hospital placed on a sound basis.

A committee arranged early this year that there should be two collections in aid of the Royal Surrey County Hospital, Guildford, and the Surrey Convalescent Home. The first collection took place in June, and was generously responded to. The second collection will take place in November.

The Duke of York paid a visit last week to the Jewish Home and Hospital for Incurables in Victoria Park Road. The President said, in his speech, that he hoped the Royal visit would result in subscriptions sufficient to erect a larger building, the foundation-stone of which he ventured to hope would be laid by the Duke of York.

The Committee of the Weston-super-Mare convalescent branch of the Bristol Hospital for Women and Children are effecting immense improvements. They have built a large play-room for the children, 38 ft. by 21 ft., and various outbuildings, such as larder, scullery, and lavatories are being constructed.

It has been resolved to hold a friendly meeting in the Castle Court-yard at Dudley on July 25th in aid of the Guest Hospital, the Dispensary, the Birmingham Eye Hospital, and the Wolverhampton Eye Hospital. the sum of £48 15s. 2½d. was raised last year by this means and divided between the above institutions.

We stated in our issue of July 21st that the public collections for the Haywood Hospital and North Staffordshire Infirmary were made on the last Saturday in June. The Clerk to the Governors writes to say that the Haywood Hospital did not benefit by the collections in any way, as the proceeds were for the benefit of the North Staffordshire Infirmary.

### BOOKS RECEIVED.

SIR JOSEPH CAUSTON AND SONS.
"Mountain, Moor, and Loch." Illustrated in Pen and Pencil.

W. AND R. CHAMBERS.
"Organic Chemistry." (Part I.) By W. Perkin, F.R.S., and F. J. Kipping, Ph.D.

**Periodicals and Pamphlets.**—The Charity Organization Review, Children's League of Pity Paper, Guy's Hospital Gazette, the South African Empire, the Medical Pioneer, the Westminster Review, Reich's Medicinal Anzliger, Creameries and Infectious Diseases, Amateur Gardening, the Strand Magazine, Tit-Bits, the Million, the Picture Magazine, and Illustrated Penny Tales.

### THE STUDENT OF MEDICINE.

### APPOINTMENTS.

Miss Annette Benson, appointed First Physician to the Kama Hospital, Bombay. F. A. Elkins, M.B., C.M.Edin., appointed Medical Superintendent of the Borough Asylum, Sunderland. C. G. Gibson, M.B., C.M.Edin., reappointed Medical Officer of Health to the Launceston Town Council. T. W. Griffith, M.D. Aberd., Professor of Anatomy, Yorkshire College, appointed Examiner in Anatomy at the University of Aberdeen. R. S. Hardman, L.R.C.P., M.R.C.S., appointed Junior House Surgeon to the Wigan Infirmary. A. E. Joscelyn, M.R.C.S., appointed Medical Officer to the Greenwich District of the Post Office. J. Keay, M.D., F.R.C.P.Edin., appointed Medical Superintendent of the District Asylum, Inverness. G. R. Parker, L.R.C.P.Lond., M.R.C.S.Eng., reappointed Medical Officer of Health for the Lancaster Rural Sanitary District. J. Poland, F.R.C.S., appointed Consulting Surgeon to the St. Pancras and Northern Dispensary. S. K. Smith, F.R.C.S., appointed Honorary Assistant Surgeon to the Liverpool Stanley Hospital. E. Sutton, M.R.C.S.Eng., L.R.C.P.Lond., appointed Assistant House Surgeon to the Poplar Hospital for Accidents, Blackwall, E. H. H. Weekes, M.R.C.S., L.R.C.P.Lond., appointed Medical Officer to the Gillingham Infectious Hospital. J. H. Williams, M.D., M.B., C.M.Edin., reappointed Medical Officer of Health for Flint Borough.

### VACANCIES.

**Resident Medical Officers.**

Addenbrooke's Hospital, Cambridge.—Resident House Physician. Salary £65 per annum, with board, lodging, and washing. Applications to the Secretary by July 30th.

Bath Eastern Dispensary.—Resident Medical Practitioner. Salary £100 per annum, with furnished apartments, coal, gas and domestic attendance. Applications to Colonel F. V. Eyre, R.A., Honorary Secretary, Rockville, Lansdown, Bath, by August 11th.

Chichester Infirmary.—House Surgeon. Salary £80 per annum, with board, lodging, and washing. Applications to Eugene Street, Secretary, by August 6th.

City of London Ophthalmic Hospital, 238A, Gray's Inn Road, W.C.—House-Surgeon. Rooms, coals, and light provided. Applications to the Secretary by August 7th.

Fisherton Asylum.—Assistant Medical Officer, not more than 30 years of age. Salary £100 per annum, with board, lodging, and washing. Applications to Dr. Finch, Salisbury.

Halifax Infirmary and Dispensary.—Assistant House-Surgeon, unmarried, doubly qualified. Salary £50 per annum, with residence, board, and washing. Applications to Outis Webster, Secretary, by August 1st.

Manchester Royal Infirmary.—Resident Surgical Officer; doubly qualified, unmarried, and not less than 25 years of age. Salary £150 per annum, with board and residence. Applications to W. L. Saunder, General Superintendent and Secretary, by July 28th.

Seamen's Hospital Society, "Dreadnought."—Junior House Surgeon, doubly qualified, for Branch Hospital, Royal Victoria and Albert Docks, E. Salary £50 per annum, with board and residence. Applications to P. Michelli, Secretary, Seamen's Hospital Society, Greenwich, S.E., by July 30th.

Sussex County Hospital, Brighton.—House Surgeon; doubly qualified, unmarried, and under 30 years of age. Salary £120, rising to £140 per annum, with board, residence, and washing. Applications to the Secretary by August 7th.

THE STUDENT OF MEDICINE—*continued.*

**Non-Resident Medical Officers.**

West London Hospital, Hammersmith Road, W.—Dermatologist. Applications to R. J. Gilbert, Secretary and Superintendent, by August 1st.

Charing Cross Hospital, Strand, W.C.—Assistant Surgeon; must be F.R.C.S.Eng., and reside within three miles of the hospital. Applications to the Chairman of the Committee of Selection by July 28th.

### Medical Appointments.

SEAMEN'S HOSPITAL SOCIETY, "DREADNOUGHT."

BATH-EASTERN DISPENSARY.

